# Does increased acetabular depth affect safe infra-acetabular screw placement in acetabular fracture fixation?

**DOI:** 10.1007/s00068-020-01455-5

**Published:** 2020-07-29

**Authors:** Johannes Dominik Bastian, David Riccardo Näf, Jennifer Larissa Cullmann, Marius Johann Keel, Peter V. Giannoudis

**Affiliations:** 1 Department of Orthopaedic Surgery and Traumatology, Inselspital Bern, Bern University Hospital, University of Bern, Freiburgstrasse 18, 3010 Bern, Switzerland; 2 Department of Diagnostic, Interventional and Paediatric Radiology, University of Bern, Inselspital, Bern, Switzerland; 3 Academic Department of Trauma and Orthopaedics, School of Medicine, University of Leeds, Leeds, UK

**Keywords:** Acetabulum, Fracture, Infra-acetabular screw, Depth, Quadrilateral plate, Lateral center–edge–angle

## Abstract

**Background:**

Infra-acetabular screws enhance the fixation strength in acetabular fractures with separation of both columns. Placement without iatrogenic femoral head violation is challenging.

**Purpose:**

To assess the impact of the acetabular configuration, the patients’ age and gender on safe infra-acetabulum screw insertion.

**Methods:**

In 112 patients (69 females; mean age: 34 years, range 17–88; *n* = 200 hips), the lateral center–edge angle (LCE) was measured on radiographs. Using corresponding axial CT scans the residual distance from (the lateral border) of the screw to (the medial border of) the femoral head (“Screw-to-Femoral Head distance”; “RD_SFH”) was determined. Statistical analysis was carried out using linear regression, multiple linear regression and normal distribution estimation.

**Results:**

The mean (range) LCE angle was 30° (7°–51°) and the mean (range) “RD_SFH” was 5 mm (1–14 mm). The linear regression model shows a significant linear relation between LCE and “RD_SFH” with a slope parameter of − 0.15 (*p* value < 0.0001), the Pearson correlation between LCE and “RD_SFH” is − 0.56 (CI [− 0.71, [− 0.40]). Age did not have a significant impact on the relation between LCE and “RD_SFH” (*p* value 0.85). Compared to male patients, in females, the intercept is 4.62 mm (*p* value 0.0005) less, the slope parameter is 0.09 (*p* value 0.029) larger.

**Conclusion:**

The virtual possibility to place an infra-acetabular screw was given in all patients. An increasing depth of the acetabulum correlated with a decrease in residual distances. As hip joint cartilage thickness was not considered in measurements, intraoperative rule-out of screw mispositioning especially in deep acetabular sockets and females is still of utmost importance.

## Introduction

An increasing incidence of geriatric acetabular fractures involving the anterior column according to the classification by Judet et al. [1] has been recently reported [2]. In older adults, open reduction and internal fixation is recommended for displaced fractures in patients who are fit for surgery and if acceptable, reduction can be obtained within short operating time by the use of a single approach (e.g., ilioinguinal approach, Stoppa approach) [3]. Fixation of the posterior column remains challenging in acetabular fractures with separation of both columns using a single anterior approach only.

**Fig. 1:**
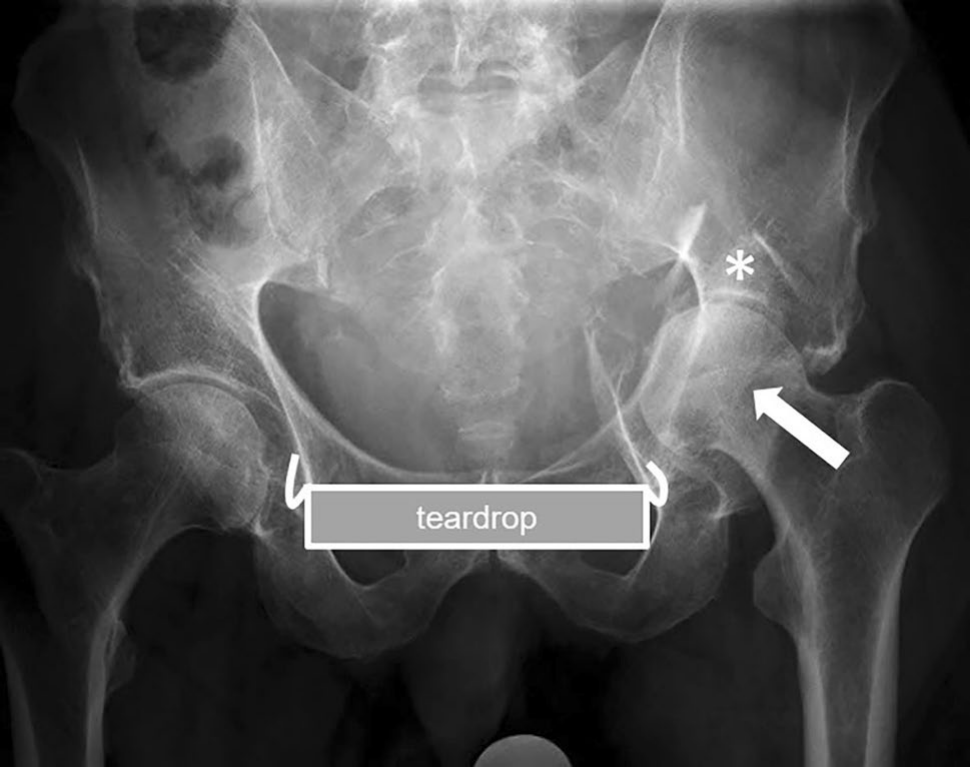
Preoperative anteroposterior radiograph of the pelvis showing an acetabular fracture (incomplete anterior column posterior hemitransverse) with break-out of the quadrilateral plate, impaction at the acetabular roof (asterisk) and medial subluxation of the femoral head in a 79-year-old male patient

**Fig. 2:**
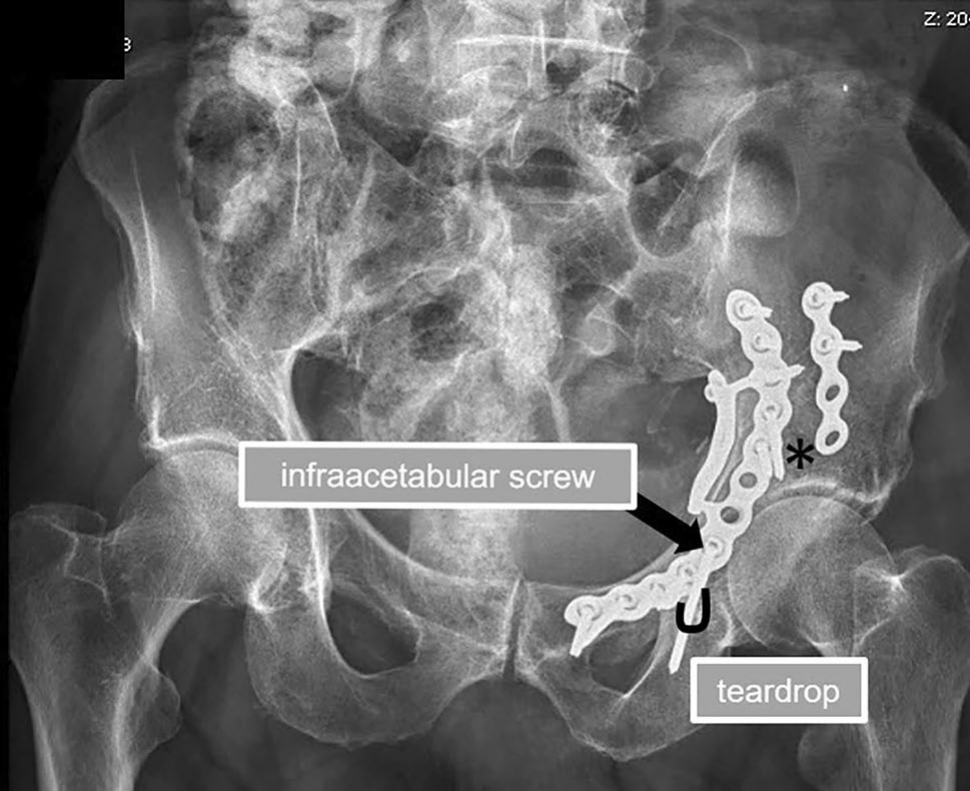
Postoperative anteroposterior radiograph of the pelvis showing an anatomic reduction (asterix) of the acetabular fracture (incomplete anterior column posterior hemitransverse) with break-out of the quadrilateral plate and impaction at the acetabular roof in a 79-year-old male patient. To enhance the fixation strength, an infra-acetabular screw (black arrow) was applied with the teardrop being the isthmus of the screw

Latest fixation techniques related to the management of these fractures include the use of infra-acetabular screw (Figs. 1, 2) and supra-acetabular screw, which facilitate closure of both columns from anterior [4]. Noteworthy, this infra-acetabular screw has shown to enhance the fixation strength in these fractures in a biomechanical study [5]. The screw is placed strictly parallel to the quadrilateral plate at the level of the teardrop [4]. Modern surgical approaches, such as the modified Stoppa approach and the Pararectus approach as anterior approaches, facilitated placement of that infra-acetabular screw in human cadavers [6].

**Fig. 3:**
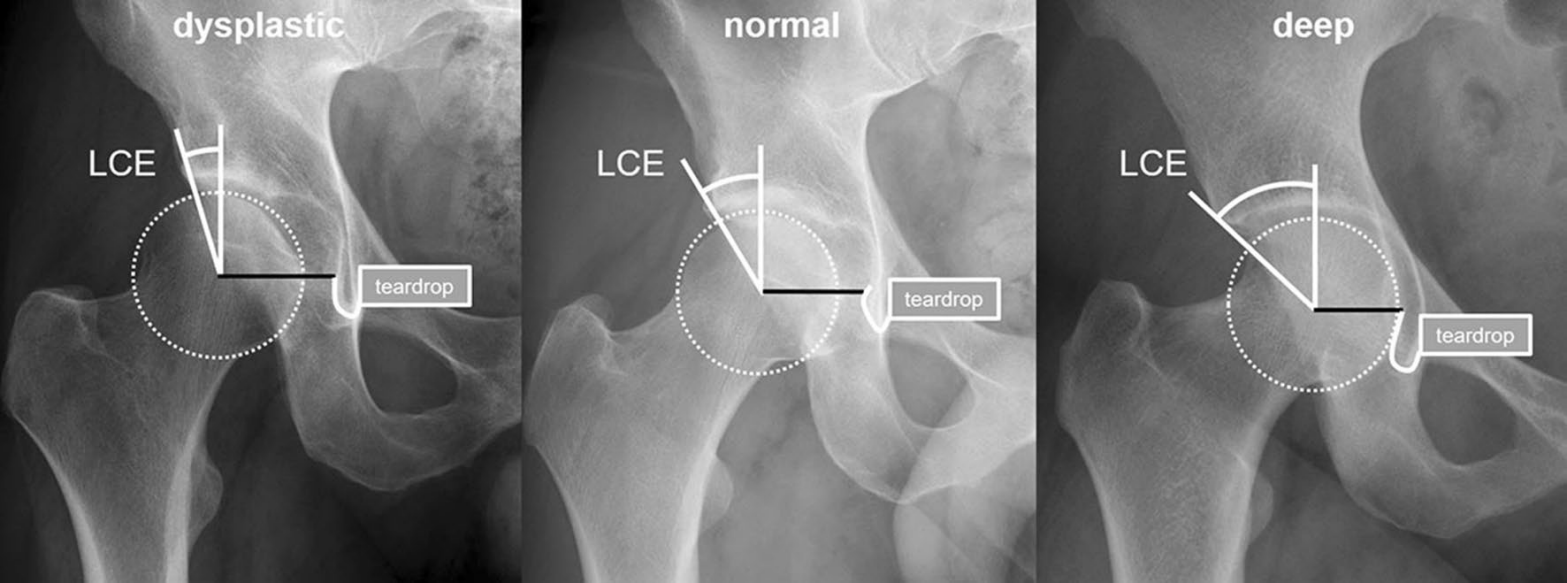
Anteroposterior radiographs of different right hip joints: the lateral center edge (LCE) angle is increasing, whilst the distance from the femoral head center to the teardrop (black line) decreases from a dysplastic to a normal and to a deep acetabular socket configuration

To our knowledge, the residual distance from the lateral border of an infra-acetabular screw to the medial border of the femoral head has not been described until now. However, a negative correlation of the minimum thickness of the medial acetabular wall with the acetabular depth was reported previously [7]. Accordingly, the medial bone stock of the quadrilateral plate might decrease with increasing depth of the acetabulum (Fig. 3), so that an infra-acetabular screw might be at risk to harm the hip joint by its placement adjacent to the femoral head. Thus, the following study questions were considered:

- Is placement of an infra-acetabular screw without violation of the femoral head in acetabular fracture fixation independent on acetabular configuration?
- Is there any impact of patients’ age and gender on the feasibility to place that screw without violation of the femoral head?
- Is it appropriate to refer on one hip to estimate the chance for screw placement of the contralateral side within an individual without violation of the femoral head?

Consequently, the aim of this study was to evaluate and correlate residual distances between the lateral border of a potential infra-acetabular screw and the medial border of the bony femoral head in different acetabular socket configurations within and between patients of different age and gender.

## Patients and methods

### Study design

Out of an existing radiographic database at our institution from 2003 to 2015, 200 hips were selected by randomization. Hips were included if CT scans and anteroposterior radiographs of the pelvis were both available and radiographs were with correct rotation and tilt [8, 9]. Hips were excluded in presence of previous surgeries, fractures or deformities. Finally, 112 patients (69 females, 62%) with a mean age of 34 years (range 17–88 years) were included; In 88 patients both (left and right) hips were measured, so that 200 measurements were performed. For patients with two measurements, one side was chosen randomly (68 left hips).

### Imaging methods

The computed tomography scans were generated by a Somatom Sensation 64 Multislice (Siemens Healthineers, Erlangen, Germany) and since 2012 by a 128-detector row CT Scanner Somatom Definition Edge (Siemens Healthineers, Erlangen, Germany) as part of the routine clinical workup. Patients were placed in the supine position on the CT table. The image resolution was 512 × 512 pixels; slice thickness was 0.6 mm–1 mm.

### Assessments

**Fig. 4:**
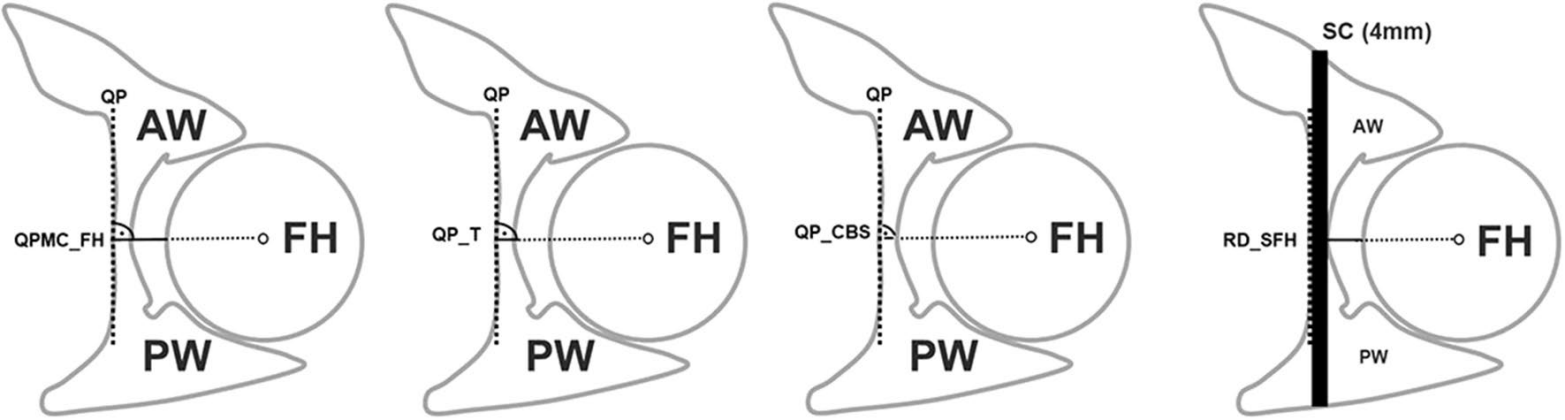
Schematic drawings illustrate the performed measurements of a left hip joint at the level of the center of the femoral head and the teardrop using axial reconstructions of a computed tomography scan (anterior wall: AW, posterior wall: PW, femoral head: FH). The following distances were measured perpendicular to a line (black dotted line) tangentially to the medial cortex of the quadrilateral plate (QP) at the level of the femoral head center: QP medial cortex to femoral head (QPMC_FH), QP thickness (QP_T) and QP cancellous bone stock (QP_CBS). Schematic drawing showing a potential screw corridor (SC; 4 mm in diameter) for a 3.5-mm cortical screw; the residual distance from **(**the lateral border of) the screw to (the medial border of) the femoral head (RD_SFH) was calculated as follows: ${\text{RD}}\_{\text{SFH}} = \, \left( {{\text{QPMC}}\_{\text{FH}}} \right) {-} \frac{{\left( {{\text{QP}}\_{\text{T}}} \right) \, {-} \, \left( {{\text{QP}}\_{\text{CBS}}} \right)}}{2} - 4{\text{ mm}}$

On anteroposterior radiographs, the lateral center–edge angle (LCE angle) was measured by the angle formed by a line parallel to the longitudinal pelvic axis and a line connecting the center of the femoral head with the lateral edge of the acetabular sourcil as reported earlier [10, 11] and as shown in Fig. 3. On CT scans using axial reconstructions at the level of center of the femoral head and the teardrop, the following distances were measured: (1) “Quadrilateral plate medial cortex to femoral head” (QPMC_FH) (B) “Quadrilateral plate thickness” (QP_T) and (C) “Quadrilateral plate cancellous bone stock” (QP_CBS) as shown in Fig. 4. The residual distance from (the lateral border of) the screw to (the medial border of) the femoral head—named as “Screw-to-Femoral Head distance” (RD_SFH)—was then calculated (Fig. 4). An originally uninvolved observer (D. R. N.) performed all measurements. We confirm that this study respects the ethical standards in the Helsinki Declaration of 1975, as revised in 2000, as well as national law.

### Statistical analysis

A linear regression model was used to investigate the relationship between LCE angle and the residual distance to the femoral head (RD_SFH). The Pearson correlation and its 95% confidence interval were calculated by dividing the slope parameter by the standard deviation of distance and multiplied by the standard deviation of LCE. Furthermore, a multiple linear regression model was used to investigate the influence of age and gender on the relationship between LCE angles and „RD_SFH”. To analyze whether or not the LCE angle of one hip is capable to predict the “RD_SFH” of the contralateral hip within one individual, additional linear regression models were performed. A pairwise *t* test was performed to see, if there is a difference in mean between the left and right LCE angle. To predict the LCE angle of one hip by the “RD_SFH” of the contralateral hip within one individual, a normal distribution with mean zero was modeled and a 95% prediction interval was calculated. All analyses were performed with R Core Team [12].

## Results

The measured mean LCE angle was 30° (range 7°–51°). The mean calculated “RD_SFH” was 5 mm (range 1–14 mm). The linear regression model shows a significant linear relation between LCE and “RD_SFH” with a slope parameter of − 0.15 (*p* value: < 0.0001; Table 1); the Pearson correlation between LCE and “RD_SFH” is − 0.56 (CI [− 0.71, − 0.40]).

**Table 1:** Regression showing statistical significance indicating a decrease of about 1.5 mm in the residual distance screw to femoral head (“RD_SFH”) if the LCE angle increases by 10°

Estimate	Value	Std. error	*p* value
Intercept	10.12	0.67	< 0.0001
LCE	− 0.15	0.02	< 0.0001

**Fig. 5:**
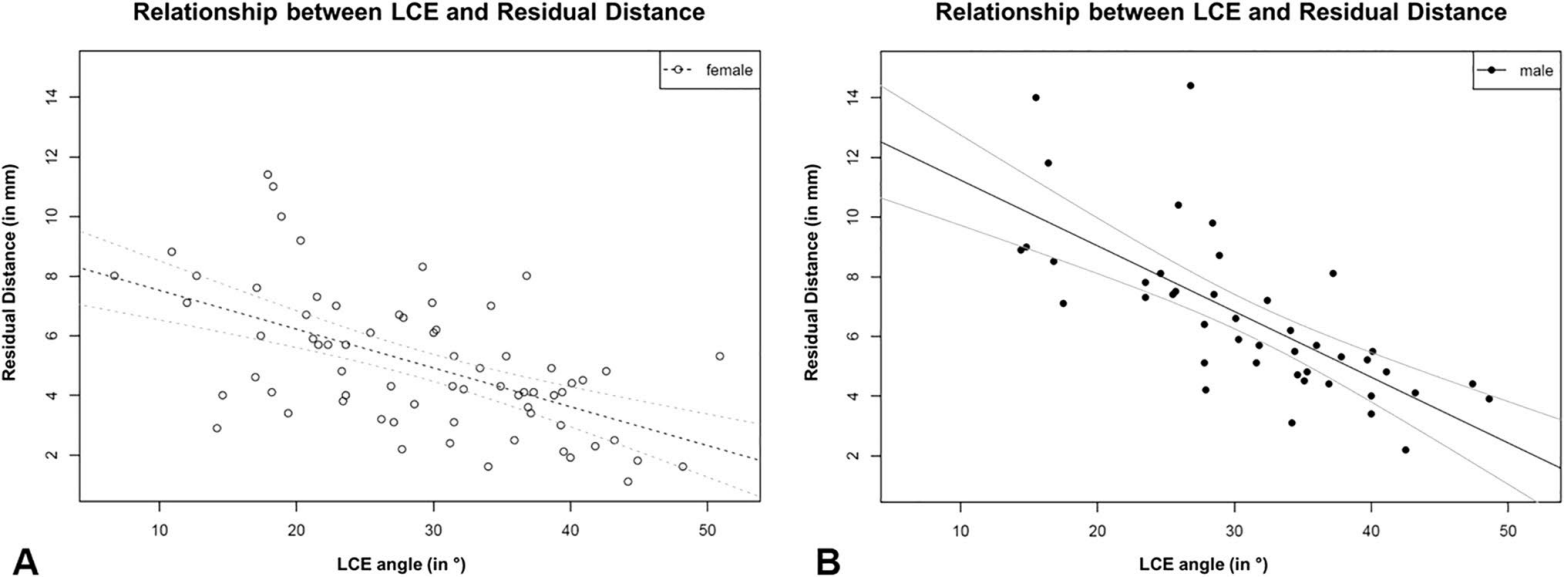
The plot shows the scatterplot between LCE angle (in °) and the residual distance (RD_STF; in mm), the fitted regression line (black) and the pointwise 95% confidence interval (gray lines) for **a** females and **b** males. In both plots, no residual distances below 0 mm were noticed; with an increasing LCE angle, the residual distances decrease

**Fig. 6:**
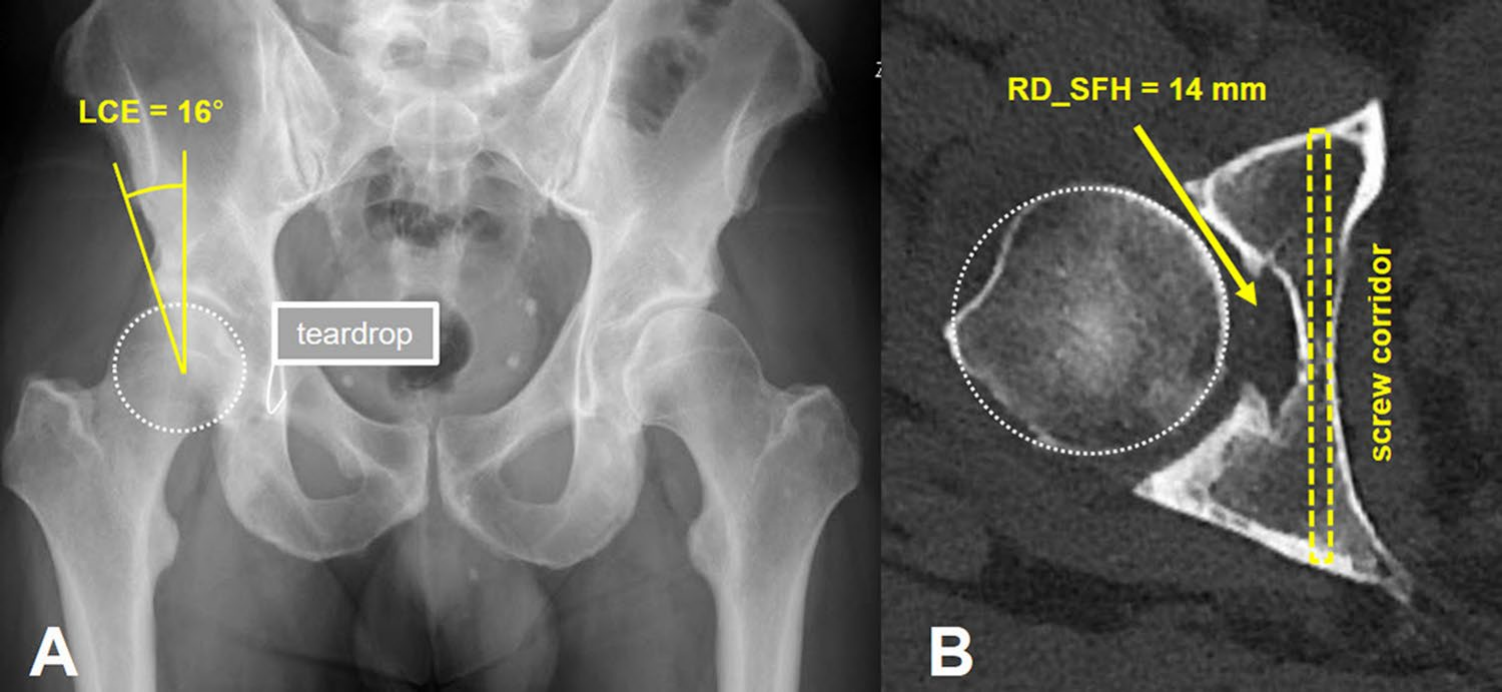
Example illustrating the relationship of **a** a low lateral center edge angle (LCE: 16°) in the right hip joint on an anteroposterior radiograph and **b** the corresponding high “virtual” residual distance from the lateral border of an infra-acetabular screw to the medial border of the femoral head (RD_SFH: 14 mm) as measured on CT

**Fig. 7:**
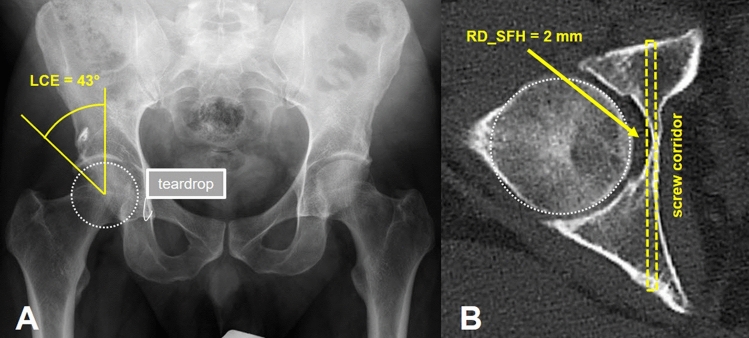
Example illustrating the relationship of **a** a high lateral center edge angle (LCE: 43°) in the right hip joint on an anteroposterior radiograph and **b** the corresponding low “virtual” residual distance from the lateral border of an infra-acetabular screw to the medial border of the femoral head (RD_SFH: 2 mm) as measured on CT

The multiple linear regression model with the independent variables LCE, gender and their interaction provides significant results. Age does not have a significant impact on the relation between LCE and “RD_SFH” (*p* value 0.85) and is, therefore, not considered. The intercept for male patients is 13.44 mm and the intercept for female patients is 8.82 mm, − 4.62 mm (*p* value 0.0005) less than for male patients. The slope parameter for male patients is − 0.22 (*p* value < 0.0001) and the slope parameter for female patients is − 0.13, it is 0.09 (*p* value 0.029) larger than for male patients. Accordingly, in case of a LCE angle of 0°, the “RD_SFH” measures 13.44 mm in males, an increase of the LCE angle of 10° decreases the “RD_SFH” by 2.2 mm on average. In case of a LCE angle of 0°, the “RD_SFH” measures 4.62 mm less in females than in males, an increase of the LCE angle of 10° decreases the “RD_SFH” by only 1.3 mm on average (Table 2). The scatterplot between LCE angle (in °) and the residual distance (RD_STF; in mm), the fitted regression line (black) and the pointwise 95% confidence interval (gray lines) are shown in Fig. 5. No residual distances below 0 mm were noticed; with decreasing LCE angles, the residual distances increase and vice versa (examples shown in Figs. 6, 7).

**Table 2:** Multiple Regression analysis showing statistical significance indicating a decrease of about 2.2 mm for male and 1.3 mm for female in the residual distance screw to femoral head (“RD_SFH”) if the LCE angle increases by 10°

Estimate	Value	Std. error	*p* value
Intercept	13.44	1.08	< 0.0001
LCE	− 0.22	0.03	< 0.0001
Female	− 4.62	1.3	0.0006
LCE: female	0.09	0.04	0.03

**Fig. 8:**
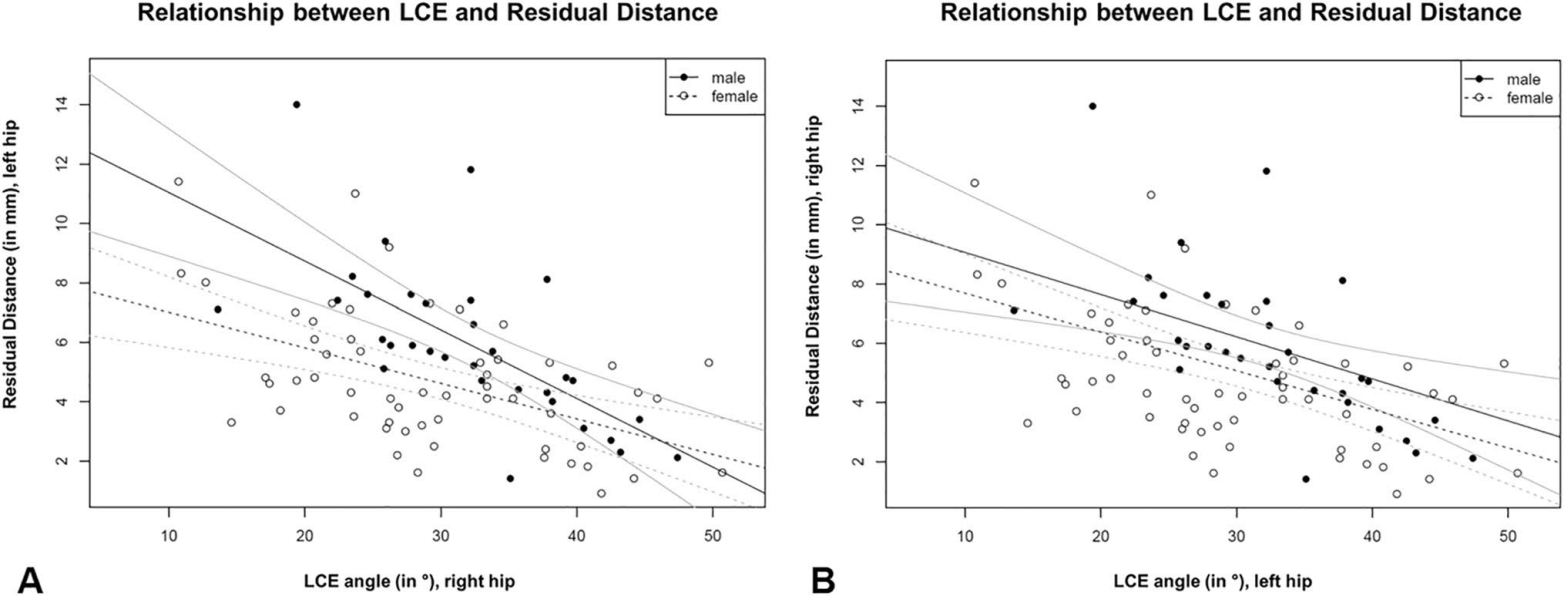
The plot shows the scatterplots between **a** LCE angle (in °) of the right hip and the residual distance (RD_STF; in mm) of the left hip and **b** LCE angle (in °) of the left hip and the residual distance (RD_STF; in mm) of the right hip with fitted regression lines (black) and pointwise 95% confidence intervals (gray lines) for females and males

The scatterplots between LCE angle (in °) of one hip and the residual distance (RD_STF; in mm) of the contralateral hip and the fitted regression line (black) and the pointwise 95% confidence interval (gray lines) are shown in Fig. 8. Visually, the regression lines in Fig. 8a and b are similar. This indicates that the LCE angle of one hip is capable to predict the “RD_SFH” of either side.

**Fig. 9:**
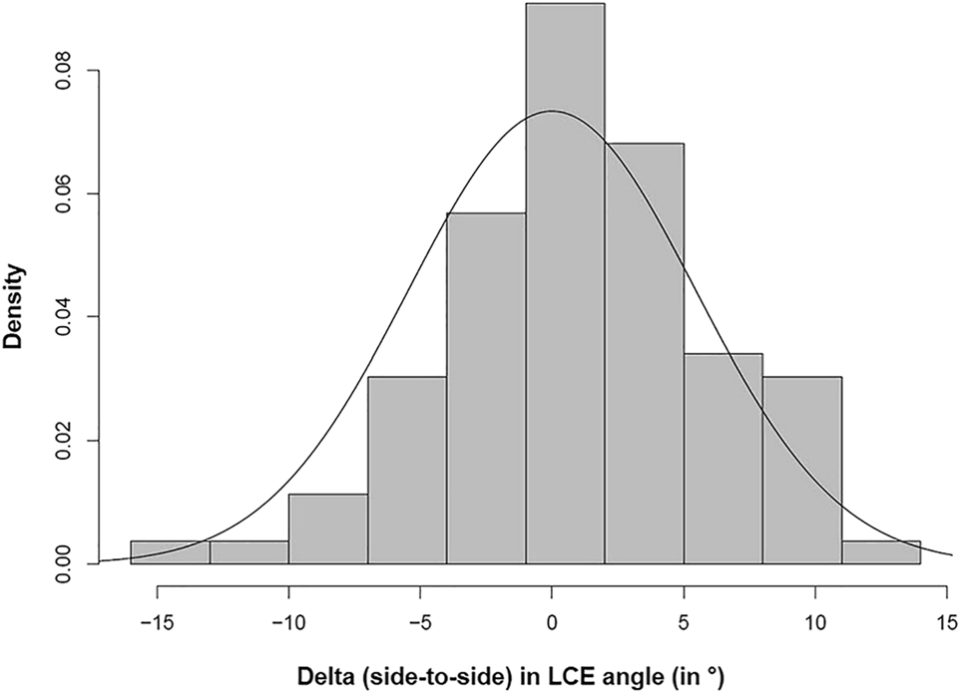
The plot shows a histogram of the measured differences between the LCE angle on the left side and the LCE angle on the right side. The estimated normal density function (black) with mean zero and estimated standard deviation 5.4 shows that the model fits the data well. The 95% prediction interval for the difference between the LCE angle of one hip and the LCE angle of the contralateral hip within one individual is then given by [− 10.8, 10.8]

The measured differences between the LCE angle on the left side and the LCE angle on the right side are shown in a histogram in Fig. 9. A pairwise *t* test shows no significant difference in mean (Estimated difference: 1.1 mm; *p* value: 0.057). Therefore, a normal density function (black) with fixed mean zero and estimated standard deviation 5.4 was fitted. The model fits the data well. The 95% prediction interval for the difference between the LCE angle of one hip and the LCE angle of the contralateral hip within one individual is then given by [− 10.8, 10.8].

## Discussion

Geriatric acetabular fractures have been increasing rapidly; whereas, traditional hip fractures declined significantly within the last 20-year period [2, 13, 14]. These fractures represent a surgical challenge, as fixation has to be as rigid as possible to increase fixation strength in osteoporotic bone quality in patients mainly unable to comply with non-weight bearing instructions postoperatively.

The literature provides evidence that an infra-acetabular screw is a sufficient modification of standard techniques independent on the used plate systems and obtainable everywhere as a low-cost opportunity to enhance fixation strength in geriatric acetabular fractures [5, 15]. In contrast, the challenge of secure infra-acetabular screw placement is the subject of current studies. These aim on morphometrical analyses to describe potential infra-acetabular screw corridors and, thus, express the need to enhance the safety of infra-acetabular screw placement [16–18].

Gras et al. described in a biomorphometric analysis that a bony corridor with a diameter of at least 5 mm for secure infra-acetabular screw placement could be determined in 93% of cases [16]. However, these results do not provide information about the dimension of the cancellous infra-acetabular corridor as the infra-acetabular screw projects medially to the cortical bone of the acetabular fossa and laterally to the cortical bone of the obturator canal with the teardrop being the isthmus [4]. In addition, pelves with hip dysplasia were excluded. In contrast, Arlt et al. assessed the intraosseous secure screw corridor dimensions without exclusion of dysplastic hips with a computer-assisted 3D radiomorphometric analysis [18]. A corridor for a 3.5-mm screw was present in 94% of all cases, and the teardrop diameter was considered a safety indicator with cut-off values of more than 4 mm as measured on radiographs in anteroposterior view of the pelvis; a more capacious quadrilateral bone stock was not identified in the presence of dysplasia. However, the methodological workflow used in that study is not feasible for applications in daily routine clinical practice [18]. Baumann et al. assessed anatomical landmarks and specific entry points rather than corridor dimensions [17]. In summary, these previous studies reported about infra-acetabular corridor dimensions and anatomic landmarks to enhance intraoperative orientation rather than enhancing preoperative planning using standard parameters regularly seen on plain radiographs of the pelvis.

To our knowledge, the herein study assessed for the first time the relation between the acetabular configuration (with focus on a deep acetabulum) expressed by standard parameters (LCE angle). Others used the LCE angle previously to clarify its relationship to the thickness of the medial acetabular wall to assist orthopedic surgeons in planning total hip replacements [11] or to describe a deep acetabular socket [19, 20]. In addition, the relation of the residual distance between the femoral head and screws placed within an infra-acetabular corridor was not described previously; however, knowledge about these residual distances might guide more appropriately to avoid screw penetration in the hip joint.

The results of our study show that secure placement of an infra-acetabular screw in acetabular fracture fixation is dependent on the acetabular configuration. The residual distances decrease with increasing LCE angles and vice versa. For dysplastic hips, the results are in accordance with a previous report showing a thicker medial acetabular wall [11]. However, Arlt et al. could not confirm this observation and consequently, one could speculate that differences in patient selection and definition of dysplasia might account for this [18]. Our results further show that there is no impact of age but rather of gender on the feasibility to place an infra-acetabular screw securely. In females, residual differences were overall shorter compared to males, a finding which is in accordance with Gras et al. who observed gender-specific differences in infra-acetabular corridors (female corridors were significantly smaller in size (diameter and length), and the axis more angulated compared with male corridors) [16]. Based on our results, the LCE angle of one (e.g., uninjured) hip might be used to calculate the residual distance between an infra-acetabular screw and the femoral head of the contralateral (fractured) hip joint.

A limitation of our study and also of previous reports might be that measurements were performed using CT scans so that evaluated residual distances “femoral head-to-screw” are based on bony landmarks only. Thus, conclusions based on our measurements might be limited. Femoral head cartilage thickness was measured in cadavers previously with a median thickness of about 3 mm [21]. Accordingly, in patients with residual distances of less than 3 mm, screw placement might be at risk to damage femoral head cartilage. In addition, following different reasons (e.g., osteoporotic bone) fracture fragments might migrate until fracture union and the femoral head might slightly re-displace medially and then the screw might harm the femoral head. Another concern might be the observed variation at smaller residual distances. This variation might be caused by differences in the width of the joint space due to differences in cartilage thickness. In that context, a limitation might be that the amount of a “sufficient distance” to protect the femoral head is not reported. Another limitation might be that LCE angles were measured in uninjured hip joints that might be unfeasible in fractures of the acetabulum with medial protrusion of the femoral head. However, we performed a LCE angle “side-to-side” analysis which showed a mean “side-to-side” difference of only 1° that might not be relevant in clinical practice. The LCE angle of the contralateral uninjured hip might assist in estimation of the residual distance in the injured hip. Another option to overcome the shortcoming above could be to measure the LCE angle after fracture reduction at the injured hip joint using the anteroposterior view of the pelvis obtained by intraoperative fluoroscan. To further exclude intraarticular misplacement, the projection of the screw as a dot in the teardrop could be used, whilst individual planning using preoperative CT scans is still mandatory [16]. Another limitation might be that measurements were performed from radiographs obtained in a patient cohort without any actual screw placement so that only an estimated “virtual” possibility to place a screw can be reported. Accordingly, no information was obtained about any other significant issues (e.g., soft tissues in obesity) precluding secure placement of an infra-acetabular screw despite the presence of an adequate bony corridor.

Strengths of our study include: (1) the measurement of the true residual distance from the lateral border of a potential infra-acetabular screw to the medial border of the femoral head rather than dimensions of potential screw pathways, (2) not only normal and dysplastic but also deep acetabular configurations were included in the analysis, (3) the application of a commonly in use radiographic parameter (LCE angle) enhanced feasibility, (4) a side-to-side analysis was performed to clarify if measurements performed on the uninjured hip joint are appropriate to be used for reference for the fractured side and (5) measurements were performed at the level of the acetabular fovea (the isthmus of the screw corridor) tangential to the quadrilateral plate in direction of infra-acetabular screws.

## Conclusion

The virtual possibility to place an infra-acetabular screw was given in all patients in our study setup independent of the acetabular socket configuration, age or gender. The contralateral uninjured hip might be used for reference in preoperative planning. An observed variation at smaller residual distances especially within females might be of concern in clinical practice just as the limitation that hip joint cartilage thickness was not considered in our measurements using CT scans only. Therefore, individual preoperative planning and intraoperative rule-out of screw mispositioning is still of utmost importance, especially in the deep acetabulum and in females.
